# Supplementary material for: Genome-Wide Characterization of PEBP Gene Family and Functional Analysis of TERMINAL FLOWER 1 Homologs in Macadamia integrifolia
Source: Plants (Basel). 2023 Jul 19;12(14):2692. doi: 10.3390/plants12142692 (PMC10385423; doi:10.3390/plants12142692)
Supplement: Supplementary file 1 [file plants-12-02692-s001.zip › plants-2464194-supplementary.pdf]

Figure S1. Identification of the T1 transgenic *Arabidopsis* of *35S::MiTFL1*

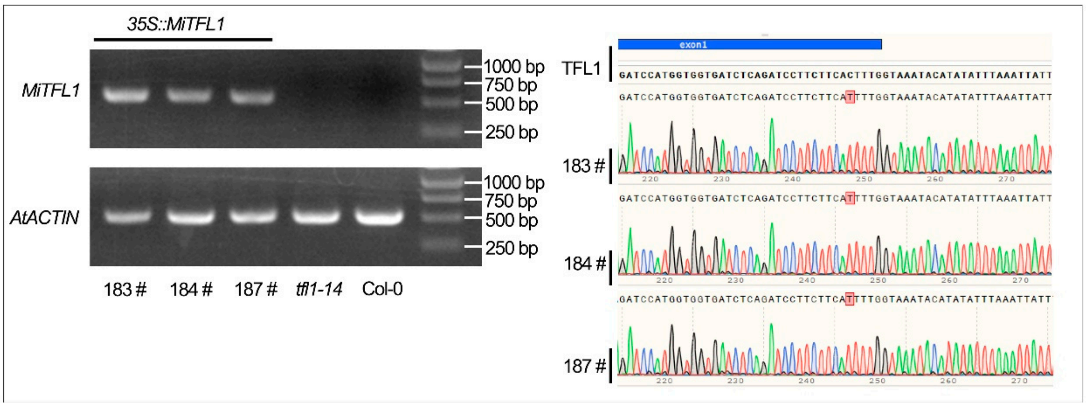

Table S1. PEBP gene family accession number of *Macadamia integrifolia* and other species.

**Species**	**Notation**	**Gene accession number from NCBI**
Macadamia integrifolia	*MiMFT1*	XM_042646923.1
	MiMFT2	XM_042664833.1
	MiMFT3.1	XM_042653947.1
	MiMFT3.2	XM_042653954.1
	MiFT1.1	XM_042626919.1
	MiFT1.2	XM_042626920.1
	MiFT2	XM_042638310.1
	MiFT3.1	XM_042647763.1
	MiFT3.2	XM_042647762.1
	MiFT4	XM_042661046.1
	MiFT5	XM_042661118.1
	MiBFT1	XM_042650477.1
	MiBFT2	XM_042655061.1
	MiBFT3	XM_042655088.1
	MiTFL1-1	XM_042633609.1
	MiTFL1-2	XM_042638431.1
Glycine max	*Dt1/GmTFL1b*	ADF30892.1
Medicago truncatula	*MtTFL1*	XM_003625760.4
Solanum pennellii	*SP*	NM_001247045.2
Antirrhinum majus	*CEN*	S81193.1
Actinidia chinensis var. chinensis	*AcBFT2*	KX611601.1
**Species**	**Notation**	**Gene accession number from Phytozome**
Arabidopsis thaliana	*AtFT*	At1g65480.1

	AtTSF	At4g20370.1
	AtTFL1	At5g03840.1
	AtBFT	At5g62040.1
	AtMFT	At1g18100.1
	AtATC	At2g27550.1
Malus×domestica	*MdMFT*	MD06G1229900
	MdTFL1	MD14G1021100
	MdCEN2	MD03G1143000
	MdBFT1	MD01G1198400
	MdTFL2	MD12G1023900
	MdFT	MD12G1262000
	MdBFT2	MD07G1265900
	MdCEN1	MD11G1163500
Oryza sativa	*OsRCN4*	LOC_Os04g33570.1
	OsFTL6	LOC_Os04g41130.1
	OsFTL1	LOC_Os01g11940.1
	OsFTL8	LOC_Os01g10590.1
	OsFTL9	LOC_Os01g54490.1
	OsMFT2	LOC_Os01g02120.1
	OsFTL5	LOC_Os02g39064.1
	OsRCN2	LOC_Os02g32950.1
	OsFTL13	LOC_Os02g13830.1
	OsFTL4	LOC_Os09g33850.1
	OsMFT1	LOC_Os06g30370.1
	Hd3a	LOC_Os06g06320.1
	RFT1	LOC_Os06g06300.1
	OsFTL12	LOC_Os06g35940.1
	OsRCN3	LOC_Os12g05590.1
	OsFTL7	LOC_Os12g13030.1
	OsFTL10	LOC_Os05g44180.1
	OsRCN1	LOC_Os11g05470.1
	OsFTL11	LOC_Os11g18870.1
Sorghum bicolor	*SbFT1*	Sobic.010G045100.1
	SbFT2	Sobic.003G017200.1
	SbFT3	Sobic.006G128500.1
	SbFT4	Sobic.004G206600.1
	SbFT5	Sobic.005G110406.1
	SbFT6	Sobic.002G262500.1
	SbFT7	Sobic.004G101800.1

	SbFT8	Sobic.003G295300.1
	SbFT9	Sobic.010G164200.1
	SbFT10	Sobic.009G199900.1
	SbFT11	Sobic.008G082200.1
	SbFT12	Sobic.006G047700.1
	SbFT13	Sobic.003G026600.1
	SbTFL1-1	Sobic.004G165100.1
	SbTFL1-2	Sobic.008G037300.1
	SbTFL1-3	Sobic.005G038400.1
	SbTFL1-4	Sobic.006G068300.1
	SbMFT1	Sobic.003G098800.1
	SbMFT2	Sobic.010G136300.1
Brachypodium distachyon	*BdFT1*	Bradi1g38150.1
	BdFT2	Bradi1g48830.1
	BdFT3	Bradi2g07070.1
	BdFT4	Bradi2g19670.1
	BdFT5	Bradi2g49795.2
	BdFT6	Bradi3g08890.1
	BdFT7	Bradi3g48036.1
	BdFT8	Bradi4g35040.2
	BdFT9	Bradi4g39730.1
	BdFT10	Bradi4g39750.1
	BdFT11	Bradi4g39760.1
	BdFT12	Bradi5g14010.1
	BdMFT1	Bradi1g42510.1
	BdMFT2	Bradi2g01020.1
	BdMFT3	Bradi2g27860.1
	BdTFL1	Bradi3g44860.1
	BdTFL2	Bradi4g42400.1
	BdTFL3	Bradi5g09270.1
Solanum lycopersicum	*SlSP6A*	Solyc05g055660.3.1
	SlSP5G	Solyc05g053850.3.1
	SlSP5G1	Solyc11g008660.1.1
	SlSP5G2	Solyc11g008640.1.1
	SlSP5G3	Solyc11g008650.1.1
	SlSP3D	Solyc03g063100.2.1
	SlSP9D	Solyc09g009560.2.1
	SlSP2G	Solyc02g079290.3.1
	SlMFT	Solyc03g119100.2.1

	SP	Solyc06g074350.3.1
	SlCEN1.1	Solyc03g026050.3.1
	SlCEN1.2	Solyc01g009580.2.1
	SlCEN1.3	Solyc01g009560.2.1
Vitis vinifera	*VvTFL1B*	VIT_208s0007g03450.1
	VvFT	ABF56526.1 (from NCBI)
	VvMFT-like	VIT_217s0000g02630.1
	VvTFL1C	VIT_216s0100g00700.1
	VvMFT	VIT_200s0203g00080.1
	VvTFL1A	VIT_206s0080g00290.1
Zea mays	*ZmZCN1*	Zm00001d044705_T001
	ZmZCN2	Zm00001d050649_T001
	ZmZCN3	Zm00001d023420_T001
	ZmZCN4	Zm00001d003804_T001
	ZmZCN5	Zm00001d025346_T001
	ZmZCN6	Zm00001d052537_T001
	ZmZCN7	Zm00001d038725_T001
	ZmZCN8	Zm00001d010752_T001
	ZmZCN9	Zm00001d008446_P004
	ZmZCN10	Zm00001d040113_P001
	ZmZCN11	Zm00001d037439_T001
	ZmZCN12	Zm00001d043461_T001
	ZmZCN13	Zm00001d016253_T001
	ZmZCN14	Zm00001d008939_T001
	ZmZCN15	Zm00001d036242_T001
	ZmZCN16	Zm00001d017134_T001
	ZmZCN17	Zm00001d004630_T001
	ZmZCN18	Zm00001d006116_T001
	ZmZCN19	Zm00001d025737_T001
	ZmZCN20	Zm00001d024023_T001
	ZmZCN21	Zm00001d004010_T001
	ZmZCN24	Zm00001d021135_T001
	ZmZCN25	Zm00001d003226_T001
	ZmZCN26	Zm00001d046300_T001

Table S2. List of primers.

Primers name	Template strand(5'-3')	Usage
tfl1-F1	GATGTCTCGGTCGTCTCTTTGTC	Sequencing identifies the *tfl1-14* mutant background

tfl1-R1	CGGATTCAACTCATCTTTGGCAG	
MiTFL1-like-F1	TTCCAAACCCAATAAGATAACC	*MiTFL1-like cloning*
MiTFL1-like-R1	ACCCTTACTCTGACGACCATAC	
MiTFL1-F1	CTCCAAGACCTATACCAACTCA	*MiTFL1 cloning*
MiTFL1-R1	CTAACTAGCCAGCTTCAACATC	
35S-MiTFL1-like-GFP-F	tttggagaggacacgctcgagATGGCAAGAACATTAGAGCCTC	*Construction of 35S::MiTFL1-like-GFP recombinant vector*
35S-MiTFL1-like-GFP-R	cttctcccttacccatggtaccACGTCTTCTTGCAGCTGTTTC	
35S-MiTFL1-GFP-F	tttggagaggacacgctcgagATGGCAAGAACATCAGAGCCTC	*Construction of 35S::MiTFL1-GFP recombinant vector*
35S-MiTFL1-GFP-R	cttctcccttacccatggtaccACGTCTTCTTGCAGCTGTTTC	
35S-MiTFL1-like-F	cgggggactcttgaccatgGCAAGAACATTAGAGC	*Construction of 35S::MiTFL1-like recombinant vector*
35s-MiTFL1-like-R	gaaattcgagctggtcaccCTAACGTCTTCTTGCAGC	
35S-MiTFL1-F	cgggggactcttgaccatgGCAAGAACATCAGAGC	*Construction of 35S::MiTFL1 recombinant vector*
35s-MiTFL1-R	gaaattcgagctggtcaccTCAACGTCTTCTTGCAGC	
qRT-MiTFL1-like-F	TTCCTGTAGCTGCGGTCTTC	*qRT-PCR of MiTFL1-like for Macadamia integrifolia*
qRT-MiTFL1-like-R	GCGGTAGTGGATGGTCTCTC	
qRT-MiTFL1-F	GGCCTAACTAGCCAGCTTCA	*qRT-PCR of MiTFL1 for Macadamia integrifolia*
qRT-MiTFL1-R	ACTAGAAGCTTTGCTGCGGA	
qRT-*MiACTIN7*-F	GAGGAGAGGATCTGTCGTAAA	qRT-PCR of *MiACTIN7* for *Macadamia integrifolia*
qRT-*MiACTIN7*-R	GATAACAAGGAGAGGCCAAAG	
MiTFL1-like CDS-F	ATGGCAAGAACATTAGAGC	*RT-PCR of MiTFL1-like for Transgenic Arabidopsis*
MiTFL1-like CDS-R	CTAACGTCTTCTTGCAGCTG	
MiTFL1 CDS-F	ATGGCAAGAACATCAGAGC	*RT-PCR of MiTFL1 for Transgenic Arabidopsis*
MiTFL1 CDS-R	TCAACGTCTTCTTGCAGCTG	

RT-*AtACTIN2*-F	CAGTGGTCGTACAACCGGTATTG	RT-PCR of *AtACTIN2* for Transgenic Arabidopsis
RT-*AtACTIN2*-R	TGCTGTGATTTCTTTGCTCATACG	

Table S3. Days to flower.

Wt (Col-0)	tfl1-14	183#	184#	187#
27	18	26	26	43
27	18	27	27	44
28	18	29	28	45
28	19	29	28	46
28	19	30	29	47
28	20	30	29	47
29	20	31	30	48
29	21	31	30	49
29	21	32	30	49
29	21	32	31	53
30	21	33	31	53
30	23	34	32	53
30	23	34	32	55
30	23	34	33	57
31	24	35	34	59

Table S4. Number of the rosette leaves.

Wt (Col-0)	tfl1-14	183#	184#	187#
14	8	14	12	21
14	9	15	13	22
14	9	15	13	23
15	9	15	14	23
15	9	16	14	23
15	9	16	14	24
15	10	17	15	24
15	10	17	15	25
16	11	17	15	25
16	11	18	15	25
17	11	19	16	26
17	12	19	16	27
17	12	20	17	28
18	12	21	17	28
19	13	24	18	29
